# Microsurgical Reconstruction of a Cranial Defect Using a Latissimus Dorsi Flap: A Case Report

**DOI:** 10.7759/cureus.88065

**Published:** 2025-07-16

**Authors:** Arym P Preza Estrada, Alexis A Granados Flores, José L Villarreal-Salgado, Damaris E Navarro Nuño, Cesar Oropeza Duarte

**Affiliations:** 1 General Surgery, Instituto de Seguridad y Servicios Sociales de los Trabajadores del Estado (ISSSTE) "General Hospital", La Paz, MEX; 2 Surgery, Hospital General Regional No. 66, Juárez, MEX; 3 Plastic and Reconstructive Surgery, Instituto de Seguridad y Servicios Sociales de los Trabajadores del Estado (ISSSTE) "Valentín Gómez Farías Regional Hospital", Zapopan, MEX

**Keywords:** cranial reconstruction, free flap transfer, latissimus dorsi flap, microsurgery, temporal defect

## Abstract

The reconstruction of complex cranial defects, particularly in the temporal region, poses a significant surgical challenge due to both functional and aesthetic considerations, as well as the anatomical constraints of the area. We present the case of a 64-year-old female patient with an extensive cranial defect in the temporal region, secondary to a previous surgical resection. Owing to the complexity of the defect and the presence of bone exposure, a microsurgical reconstruction using a free latissimus dorsi (LD) flap was undertaken. The procedure involved harvesting the flap in the lateral decubitus position, ensuring a long, well-calibered vascular pedicle, followed by free tissue transfer and microvascular anastomosis to the superficial temporal vessels. The LD flap was chosen for its volume, versatility, and reliable vascular supply, which enabled effective coverage and restoration of the cranial contour. This case highlights the LD flap as a safe and effective option for reconstructing complex cranial defects in the temporal region. Key factors in the success of the procedure included thorough preoperative planning, microsurgical expertise, and a multidisciplinary approach. Emphasis is placed on tailoring the reconstructive strategy to the defect’s size, location, and characteristics, as well as the patient’s overall condition. Ultimately, the reconstruction of complex cranial defects requires an individualized surgical approach. The free LD flap offers a versatile and dependable solution, thanks to its large surface area, robust vascularity, and ability to adapt to 3D contours. Microsurgical techniques allow for precise vascular anastomosis, promoting flap integration and reducing the risk of complications. This case demonstrates that with careful planning and execution, excellent functional and aesthetic outcomes can be achieved.

## Introduction

Coverage defects involving a large portion of the scalp with exposed calvarial bone present a significant reconstructive challenge for plastic and microvascular surgeons. In this context, we report the case of a 64-year-old female patient with a history of atypical transitional meningioma, who underwent craniotomy with tumor cytoreduction followed by radiotherapy. Postoperative complications led to the development of a soft tissue defect in the right temporal region with exposed bone, for which a latissimus dorsi (LD) flap was used to achieve soft tissue coverage.

The LD muscle, supplied by the thoracodorsal vessels - branches of the subscapular artery - can be utilized as a muscular, musculocutaneous, or fasciocutaneous flap, either free or pedicled, and has a wide range of applications in the reconstruction of the chest, breast, head and neck, and extremities. According to the Mathes and Nahai classification, it is a type V flap, characterized by a dominant vascular pedicle with additional segmental pedicles. The primary vascular supply arises from the thoracodorsal vessels, which enter the posterior surface of the muscle in the axillary region, approximately 10 cm from its insertion. Additional segmental pedicles are provided by perforating arteries from the fourth to sixth intercostal spaces and the fourth to sixth lumbar arteries. Motor innervation is supplied by the thoracodorsal nerve, which accompanies the main vascular pedicle, while sensory innervation is derived from the dorsal branches of thoracic nerves T6 to T12 [1].

Scalp coverage defects may result from various causes, including burns, tumors, and head trauma. Several free flap options are available for scalp reconstruction, including the LD flap, the anterolateral thigh (ALT) flap, and the radial forearm flap. In this case, we focus on the LD free flap. Originally described by Tansini in 1896 as a cutaneous rotation flap pedicled in the axilla for post-mastectomy breast reconstruction, this technique gained popularity throughout Europe between 1910 and 1920 [1,2]. The use of the LD muscle or myocutaneous flap remains a highly effective solution in many scenarios, offering robust, well-vascularized soft tissue coverage over extensive areas [3].

## Case presentation

A 64-year-old female patient with a 40-year smoking history, averaging seven cigarettes per day (14 pack-years), had no history of chronic degenerative diseases. Her surgical history included an open appendectomy and a laparoscopic cholecystectomy performed 15 years earlier. She was diagnosed with an atypical transitional meningioma and underwent a right frontotemporal craniectomy with cytoreductive resection of the meningioma in May 2023. She subsequently received adjuvant radiotherapy (50 Gy).

In May 2024, a second right frontotemporal craniectomy was performed, with resection of the recurrent meningioma and placement of a prosthetic mesh. The postoperative course was unfavorable, marked by delayed wound healing and a subsequent surgical site infection. Multiple microbiological cultures were obtained, isolating extended-spectrum beta-lactamase-producing *Escherichia coli*, *Staphylococcus aureus*, and *Pseudomonas aeruginosa*. Targeted antibiotic therapy was initiated based on antibiogram results.

After resolution of the infection, the prosthetic mesh was removed, and the defect was closed using a local flap in December 2024. However, the patient later presented with loss of cutaneous coverage, resulting in exposed bone in the right temporal region. She was subsequently referred to the Plastic and Reconstructive Surgery Department. Following a comprehensive case evaluation, reconstruction with a free LD flap was planned.

Surgical procedure and postoperative course

**Figure 1:**
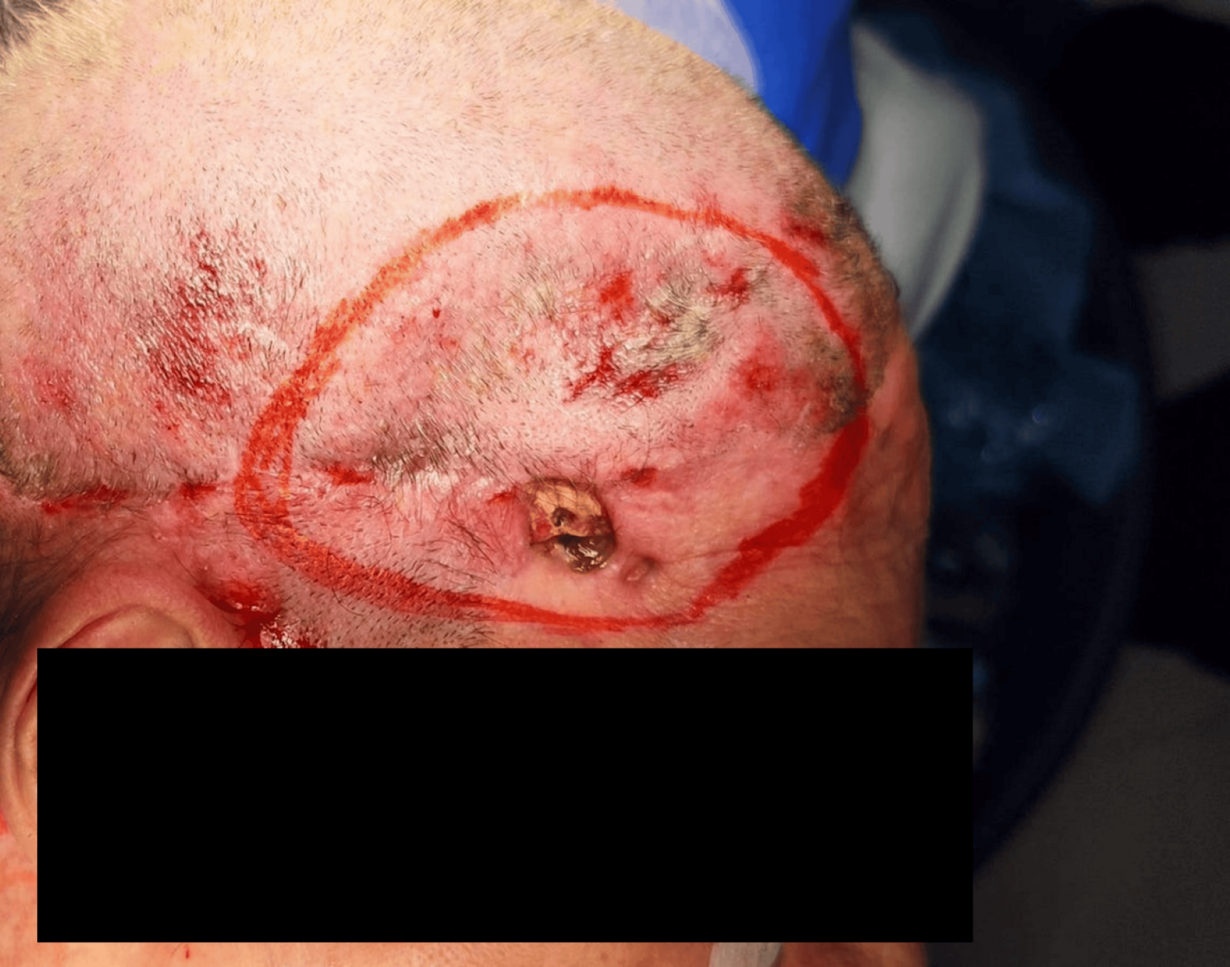
Preoperative markings The cranial defect measures 2 × 1.5 cm, with bone exposure and no signs of infection.

After induction of general anesthesia and orotracheal intubation, the surgical sites were marked (Figure 1).

**Figure 2:**
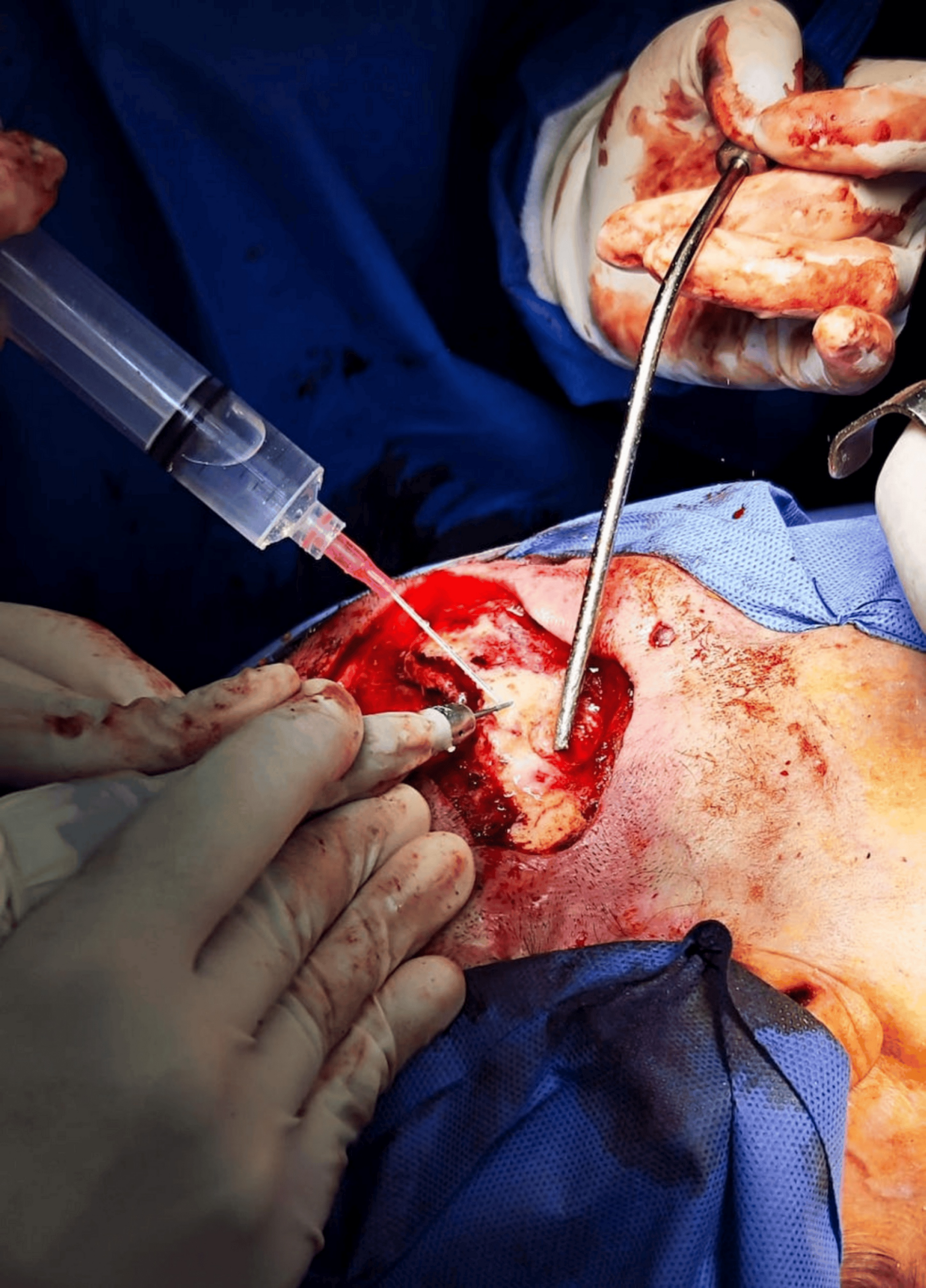
Resection of the overlying skin, subcutaneous tissue, and exposed bone

The patient was positioned in dorsal decubitus and the Rossier position. Standard aseptic and antiseptic protocols were followed to prepare the surgical field. The head was rotated to the left. A simultaneous approach was initiated at the cranial site, involving resection of the overlying skin, subcutaneous tissue, and exposed bone. The bone was then debrided and polished using a surgical drill and burr (Figure 2).

**Figure 3:**
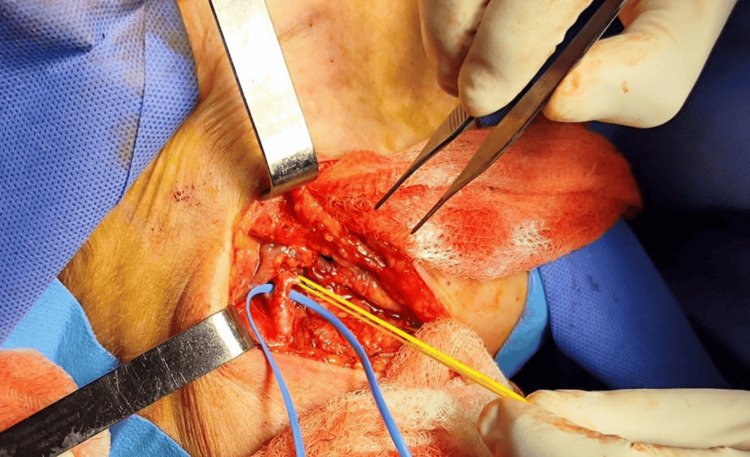
Resection of the submandibular gland to expose the external carotid artery

In parallel, a cervical approach was performed, including resection of the submandibular gland to expose the external carotid artery (Figure 3).

**Figure 4:**
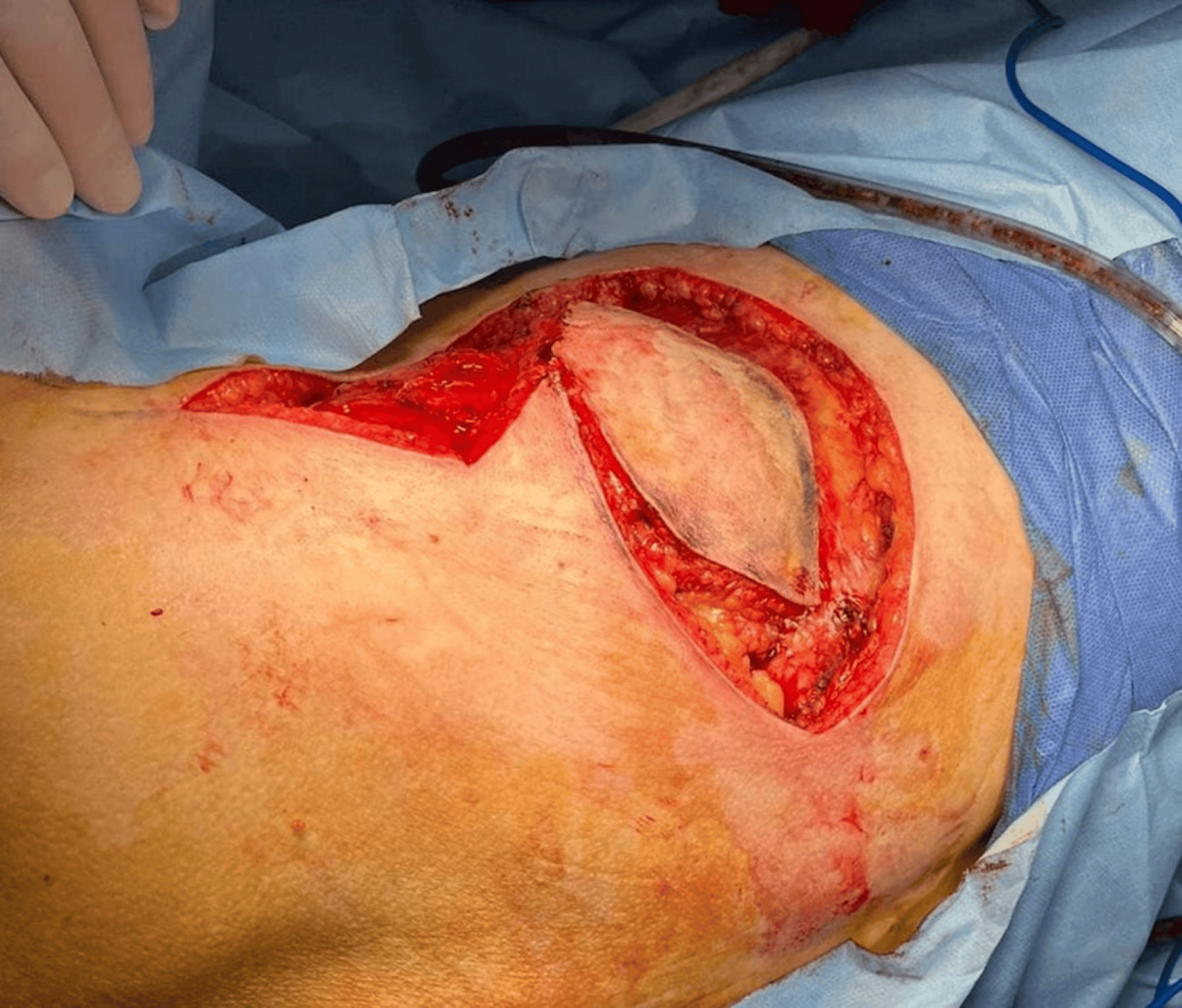
Skin island design and longitudinal incision for flap harvest

The patient was then repositioned into the left lateral decubitus position to expose the dorsal region. The skin island and longitudinal incision for flap harvest were marked and incised (Figure 4). Using transillumination, the thoracodorsal artery and vein were identified and dissected in a cephalad direction to obtain sufficient pedicle length for cranial defect coverage and microvascular anastomosis with the previously prepared cervical vessels.

**Figure 5:**
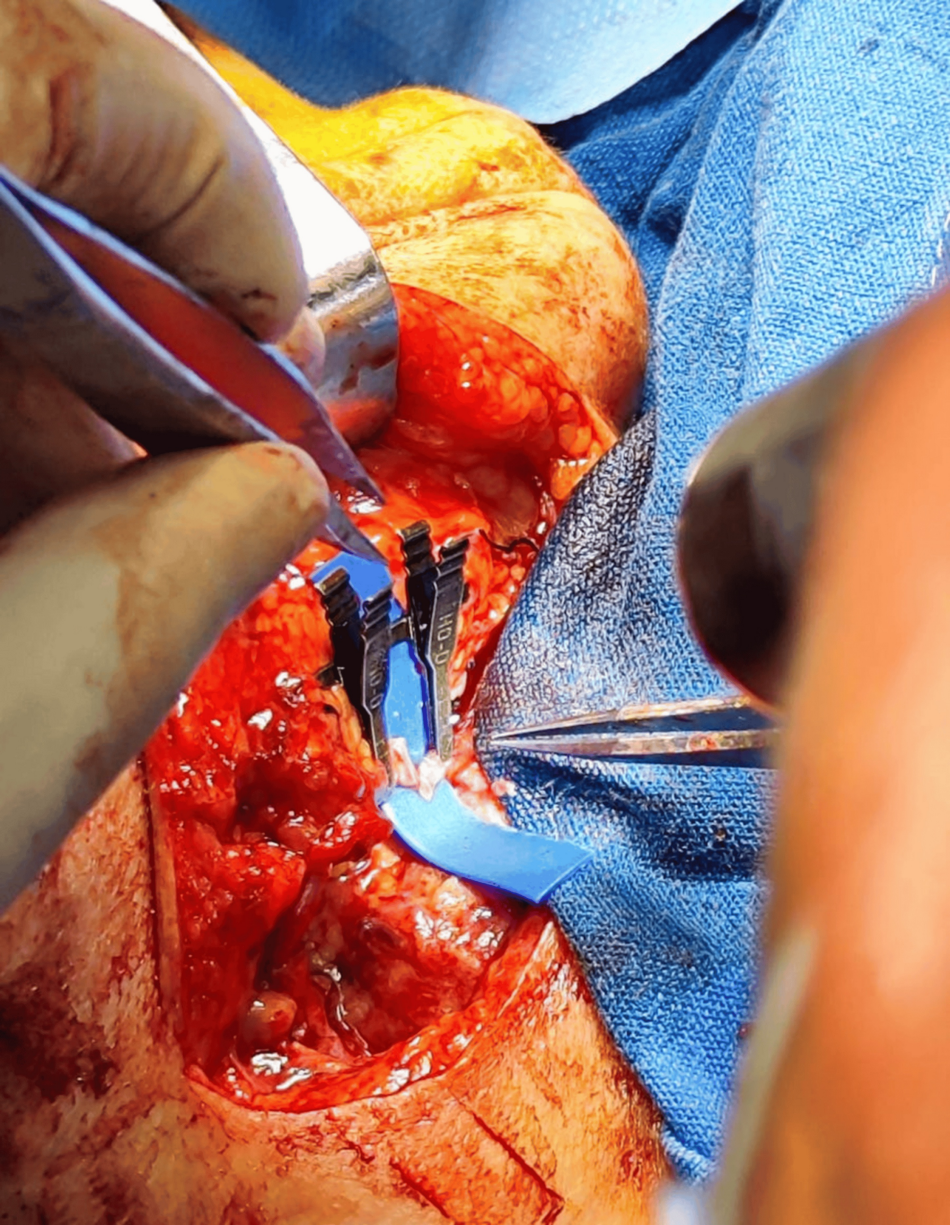
Microvascular anastomosis between the thoracodorsal artery and vein and branches of the external carotid artery, specifically the lingual artery and the inferior thyroid vein

After flap harvest, layered closure of the donor site was performed, and the patient was repositioned into the dorsal decubitus position. A subcutaneous tunnel was created between the cranial defect and the cervical approach, through which the flap was passed. It was secured with cardinal sutures. Microvascular anastomosis was performed between the thoracodorsal artery and vein and branches of the external carotid artery, specifically the lingual artery and the inferior thyroid vein, using 9-0 double-armed Prolene sutures (Figure 5). 

**Figure 6:**
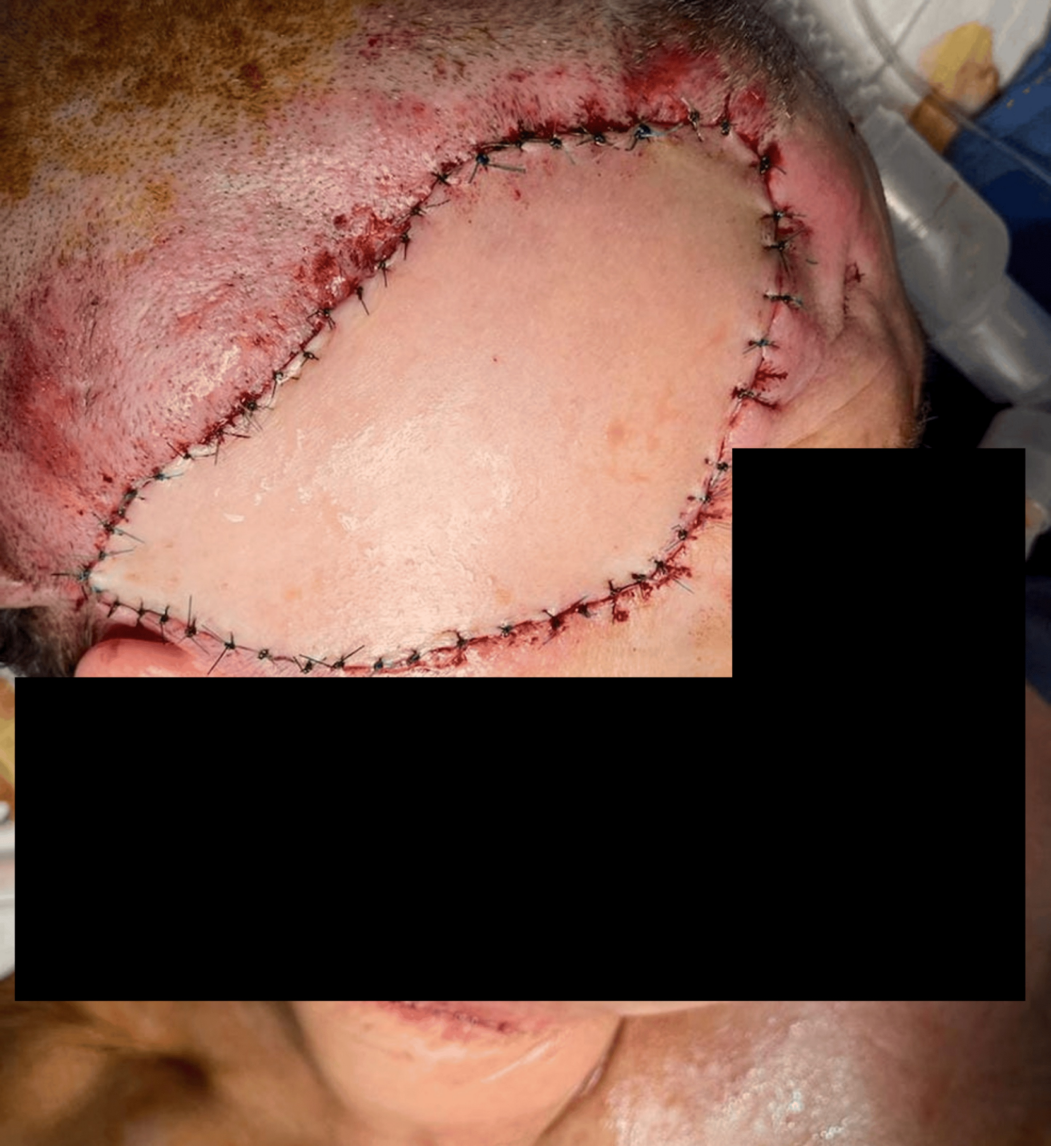
Immediate postoperative evaluation showing no signs of flap compromise

**Figure 7:**
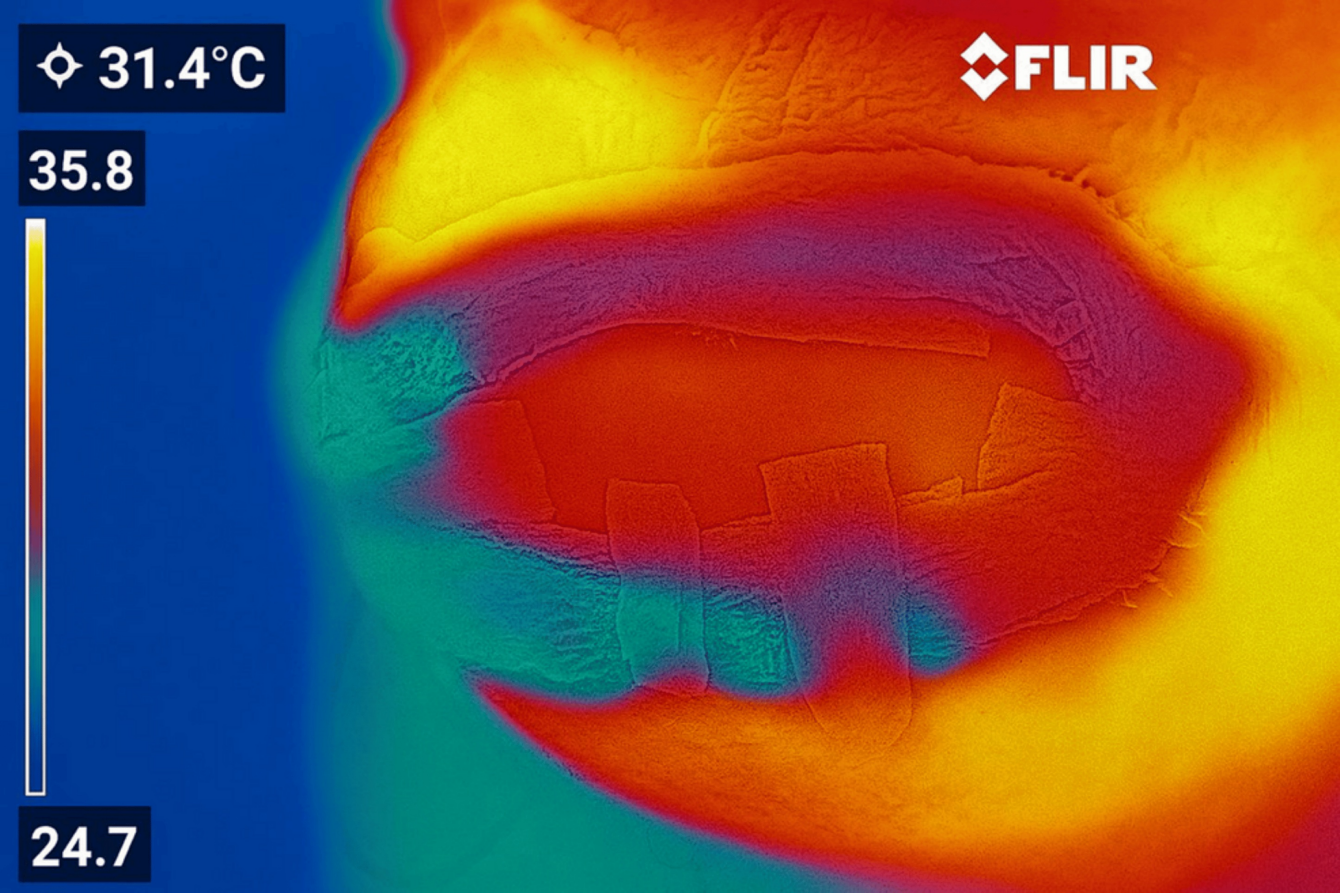
Continuous infrared thermography confirms adequate flap perfusion

Once arterial inflow and venous outflow were confirmed, the cervical incision was closed, and the flap was secured using 4-0 Prolene with interrupted simple sutures. Postoperative monitoring was initiated immediately, employing continuous infrared thermography to assess flap viability and perfusion (Figure 6, Figure 7).

## Discussion

Free flap reconstruction remains the preferred approach for addressing complex defects in the head and neck region. Among the available options, the LD flap continues to be a reliable solution, particularly for patients in whom other reconstructive methods are not viable.

In cases such as the one presented, cranioplasty may be indicated for cosmetic, protective, and therapeutic purposes. However, the implantation of cranioplasties carries a significant risk of infection, with implant exposure occurring in up to 7% of cases and overall revision rates reaching up to 27% [4]. In complex scenarios where simpler reconstructive techniques have failed, successful salvage requires interdisciplinary collaboration between neurosurgical and microsurgical teams. Scalp reconstruction is especially challenging in patients undergoing tumor excision and perioperative radiotherapy, as radiation-induced tissue changes impair wound healing and limit local flap availability.

Strübing et al. support the use of free tissue transfer for extensive and complex defects, identifying the LD free flap as one of the primary workhorses in scalp reconstruction. These cases, particularly in elderly patients, demand close collaboration between plastic surgeons and neurosurgeons [4]. Their investigation emphasizes that when free flap surgery follows osteoclastic trepanation, the entire bone defect should be covered by the skin paddle of the LD flap. This provides protection to the bone margins after muscle atrophy occurs due to denervation. Over time, the muscle thins, allowing the flap to conform to the natural convex contour of the skull, thus producing excellent long-term cosmetic outcomes. As a result, secondary thinning procedures are often unnecessary. A skin paddle over the muscle also facilitates clinical monitoring, though it can be bulky for final coverage. To address this, perforator-based monitoring of skin islands is often used [4].

The LD flap is a straightforward, reliable option that provides robust myofascial or myofasciocutaneous tissue coverage for various head and neck defects. Its favorable donor-site morbidity and the ability to employ a two-team approach make it advantageous compared to other common pedicled myofascial donor sites [5].

Since its introduction to head and neck reconstruction in 1978, the LD muscle free flap has been primarily used for large defects. Some authors describe it as the best option for such defects, as it gradually conforms to the skull’s natural contour, reducing the irregularities seen with other flap types. Moreover, its coverage potential can be expanded by including additional muscles, such as the serratus anterior, in a chimeric configuration [6].

The midface is a particularly complex anatomical region due to its 3D, prism-like structure, which must be carefully considered during reconstruction to achieve optimal aesthetic outcomes [7].

In a study by Guerrero et al., patients were evaluated weekly during the first postoperative month and monthly thereafter for nine months. Aesthetic and functional outcomes were assessed at three months post-surgery. The cosmetic evaluation considered scar appearance, body symmetry at both donor and recipient sites, and arm mobility related to the residual LD muscle. Functional evaluation focused on the recovery of movement and functionality in the treated area. He also described the use of muscle-sparing LD flaps in 13 patients, including two pediatric cases, by incorporating the internal or medial branch of the thoracodorsal artery and the superior portion of the muscle. This technique yielded favorable outcomes in terms of thoracic symmetry and LD function [7].

Numerous microsurgical techniques have been employed in the reconstruction of midface defects, utilizing various soft tissue and osseous free flaps such as the fibula, scapula, rectus abdominis, radial forearm, ALT, and LD flaps. Reported success rates range from 94% to 96%. Nevertheless, complications related to the recipient bed, donor site, and patient comorbidities can occasionally jeopardize flap survival [8].

The LD musculocutaneous flap is particularly indicated for patients who have undergone radiotherapy and require coverage of extensive defects with compromised local tissue. This flap rarely causes functional sequelae, except in highly athletic individuals or those who rely on crutches. It receives its innervation from the thoracodorsal nerve. One of the most feared complications is total flap necrosis, which presents a significant technical challenge, particularly for mastologists [9].

Overall, the LD free flap remains a versatile and dependable option for reconstructing complex scalp and cervicofacial defects, especially in previously irradiated patients or those with poor-quality local tissue. Its anatomical adaptability, low incidence of functional complications, and favorable long-term aesthetic outcomes make it a first-line choice in demanding microsurgical reconstructions. Interdisciplinary teamwork and careful patient selection remain critical for optimizing surgical success.

## Conclusions

The reconstruction of complex cranial defects in the temporal region necessitates an individualized surgical approach that ensures safe, functional, and aesthetically satisfactory coverage. The LD free flap offers a reliable and versatile solution, particularly when local tissues are compromised or unavailable. Its large surface area, dependable vascularization, and 3D adaptability make it an indispensable tool in craniofacial reconstructive surgery. Microsurgical techniques enable precise vascular anastomosis, enhancing flap integration and reducing complications. This case underscores that, with meticulous preoperative planning and technical execution, excellent functional and cosmetic outcomes can be achieved, reaffirming the essential role of microsurgery in managing highly complex defects.
